# Complete Genome Sequence of *Corynebacterium pseudotuberculosis* Strain 12C

**DOI:** 10.1128/genomeA.00759-15

**Published:** 2015-07-16

**Authors:** Thiago Jesus Sousa, Diego Mariano, Doglas Parise, Mariana Parise, Marcus Vinicius Canário Viana, Luis Carlos Guimarães, Leandro Jesus Benevides, Flávia Rocha, Priscilla Bagano, Rommel Ramos, Artur Silva, Henrique Figueiredo, Sintia Almeida, Vasco Azevedo

**Affiliations:** a Laboratory of Cellular and Molecular Genetics, Federal University of Minas Gerais, Belo Horizonte, Minas Gerais, Brazil; b Institute of Biologic Sciences, Federal University of Para, Belém, Para, Brazil; c Aquacen, National Reference Laboratory for Aquatic Animal Diseases, Federal University of Minas Gerais, Belo Horizonte, Minas Gerais, Brazil

## Abstract

We present here the complete genome sequence of *Corynebacterium pseudotuberculosis* strain 12C, isolated from a sheep abscess in the Brazil. The sequencing was performed with the Ion Torrent Personal Genome Machine (PGM) system, a fragment library, and a coverage of ~48-fold. The genome presented is a circular chromosome with 2,337,451 bp in length, 2,119 coding sequences, 12 rRNAs, 49 tRNAs, and a G+C content of 52.83%.

## GENOME ANNOUNCEMENT

*Corynebacterium pseudotuberculosis* is a Gram-positive bacterium that belongs to the CMNR group, which includes species of the genera *Corynebacterium*, *Mycobacterium*, *Nocardia*, and *Rhodococcus* (1). This species is responsible for diseases that cause great economic losses in the whole world: caseous lymphadenitis (CLA) in sheep and goats (*C. pseudotuberculosis* bv. *ovis*) and ulcerative lymphangitis in horses (*C. pseudotuberculosis* bv. *equi*) (2). With the advent of the next-generation sequencing platforms, new strains have been sequenced, and 22 strains are currently deposited in the database on the National Center for Biotechnology Information (NCBI). The increase in deposited strains can help close the pangenome this species and help the production of new vaccines and treatment methods for diseases.

Here, we present the complete genome sequence of *C. pseudotuberculosis* strain 12C. 12C was isolated from abscess of a sheep diagnosed with CLA in Pernambuco, Brazil, in 2009. This strain is suggested to belong to *C. pseudotuberculosis* bv. *ovis*, as the biochemical test for nitrate reductase showed a negative result. The genome was sequenced using the Ion Torrent Personal Genome Machine (PGM) system, 200-bp fragment library kit, and coverage of ~48-fold. The quality of the reads was analyzed using the FastQC software (http://www.bioinformatics.babraham.ac.uk/projects/fastqc), and *de novo* assembly was performed using Newbler 2.9 (Roche, USA). The assembly produced 17 contigs and an *N*_50_ value of 255,829 bp. Scaffolding was performed with CONTIGuator 2.7 (3), using as a reference the genome of *C. pseudotuberculosis* PAT10. The gap-filling process was done using the software SIMBA (http://ufmg-simba.sourceforge.net), CLC Genomics Workbench 7.0 (Qiagen, USA), and in-house scripts. Automatic annotation was performed by transferring information from a curated database using the Dinnotator software (http://lgcm.icb.ufmg.br/dinnotator). The prediction of tRNAs, rRNAs, and some coding sequences (CDSs) that were absent in the transference by Dinnotator were determined using RAST (4). All CDSs were manually curated using the Artemis software (5) and the UniProt database (http://www.uniprot.org).

The complete genome of *C. pseudotuberculosis* 12C showed a length of 2,337,451 bp in a circular chromosome, a G+C content of 52.83%, and a total of 2,119 CDSs, 12 rRNAs (5S, 16S, and 23S), 49 tRNAs, and 40 pseudogenes.

### Nucleotide sequence accession number.

This complete genome has been deposited in GenBank under the accession no. CP011474.
